# Systematic review of Acceptance and Commitment Therapy in suicide prevention in adults. Current perspective

**DOI:** 10.1192/j.eurpsy.2025.2357

**Published:** 2025-08-26

**Authors:** A. Jurado Arevalo, I. Contreras Pérez, P. Vargas Melero, M. Valverde Barea, I. Caparrós del Moral

**Affiliations:** 1 Psychiatry, University Hospital of Jaén, Jaén, Spain

## Abstract

**Introduction:**

Suicidal behavior is a public health problem in which the entire society must commit to implementing all available strategies to prevent it (Tighe et al., 2018), as more than 800,000 people die worldwide each year (Pedrola-Pons et al., 2024).

**Objectives:**

The objective is to determine the efficacy of ACT in reducing suicidal behaviors through a systematic review.

**Methods:**

A systematic review was conducted following the PRISMA 2020 methodology, searching the Cochrane, EMBASE, PubMed, PubPsych, and MEDLINE databases for scientific literature published between 2013 and March 31, 2024, using the keywords: “suicidal behavior” and “acceptance and commitment therapy” in Spanish and English. After applying inclusion and exclusion criteria, 7 studies were finally included in the systematic review.

**Results:**

After conducting the search, 7 studies were included, among which were 1 meta-analysis of randomized controlled trials, a systematic review and meta-analysis, two randomized clinical trials, a systematic review of 5 studies, a program analysis, and an effectiveness study. The review showed a significant reduction in suicidal behavior in patients who received ACT (Calati et al., 2024).

A relationship was observed between increased psychological flexibility and decreased suicidal ideation (Macri et al., 2024). The efficacy of ACT was comparable or superior to other interventions in some of the studies included in the review (Kumpula et al., 2019).

**Conclusions:**

ACT shows promising results in reducing suicidal behaviors. However, more studies with long-term follow-up are needed to confirm its efficacy.

More studies with larger sample sizes and longer participant follow-up are needed to establish the long-term efficacy of ACT in reducing suicidal behavior.

**Disclosure of Interest:**

None Declared

